# Marked Liver Tumorigenesis by *Helicobacter hepaticus* Requires Perinatal Exposure

**DOI:** 10.1289/ehp.11493

**Published:** 2008-06-13

**Authors:** Bhalchandra A. Diwan, Marek Sipowicz, Daniel Logsdon, Peter Gorelick, Miriam R. Anver, Kazimierz S. Kasprzak, Lucy M. Anderson

**Affiliations:** 1 Basic Research Program, SAIC-Frederick, Inc., Frederick, Maryland, USA; 2 Laboratory of Comparative Carcinogenesis, National Cancer Institute–Frederick, National Institutes of Health, Department of Health and Human Services, Frederick, Maryland, USA; 3 Laboratory Animal Sciences Program; 4 Pathology Histotechnology Laboratory, SAIC-Frederick, Inc., Frederick, Maryland, USA

**Keywords:** adult exposure, *Helicobacter hepaticus*, hepatocellular tumors, infection and cancer, perinatal exposure

## Abstract

**Background:**

Although severe hepatitis and liver tumors occur in a high percentage of A/J male mice naturally infected with *Helicobacter hepaticus*, these effects have not been observed after injection of adult mice with the bacteria.

**Objectives:**

We tested the hypothesis that perinatal exposure to the bacteria is required for liver tumorigenesis.

**Methods:**

A/J female mice were infected by intragastric (ig) or intraperitoneal (ip) treatment with 1.5 × 10^8^ *H. hepaticus* before pregnancy. We examined offspring at progressive time intervals, including some kept until natural death in old age. A/J, BALB/c, and C57BL/6 weanling male mice were similarly treated ig with the bacteria and observed for up to 2 years.

**Results:**

After ip bacterial infection of A/J females, 41% of their male offspring developed hepatitis and 33% had hepatocellular tumors, including 18% with hepatocellular carcinoma. Treatment by the ig route resulted in a similar incidence of hepatitis in offspring (35%) but fewer total liver tumors (8%) and carcinomas (4%). By contrast, ig instillation of *H. hepaticus* in weanling A/J, C57BL/6, or BALB/c mice resulted in low incidence of hepatitis (0–20%) and few liver tumors, despite presence of bacteria confirmed in feces.

**Conclusions:**

Results indicate that a high incidence of liver tumors in mice infected with *H. hepaticus* requires perinatal exposure. Contributing perinatal factors could include known high sensitivity of neonatal liver to tumor initiation, and/or modulation of immune response to the bacterium or its toxins. Mechanisms of human perinatal sensitivity to such phenomena can be studied with this model.

*Helicobacter hepaticus* is a murine microaerophilic bacterium first noted in association with unexplained cases of hepatitis and liver tumors in our animal colony (Ward et al. 1994b) and subsequently isolated and characterized as a new species (Fox et al. 1994). Koch’s postulates were fulfilled with regard to causation of persistent chronic hepatitis (Fox et al. 1996b). *H. hepaticus* selectively colonizes the hepatic bile canaliculi of mice, causing a morphologically distinctive pattern of chronic active progressive hepatitis, and also persistently infects the large bowel. *H. hepaticus* infection is readily established in mice by intragastric (ig) or intraperitoneal (ip) treatment with bacterial suspensions, and this method is commonly used to study the disease.

In our initial study of A/J male mice naturally infected with *H. hepaticus*, we observed hepatitis of increasing severity with age, with most mice exhibiting hepatitis by 1 year (Rice 1995; Ward et al. 1994a). Liver tumors occurred in 6% of the mice at 1 year of age, in 50% at 15 months, and in 92% at 18 months. This high incidence of liver tumorigenesis was striking, in view of human gastric cancer and lymphoma associated with infection by *Helicobacter pylori*. A later study with another sample of the naturally infected A/J males gave similar quantitative results, with liver tumors in 40% of the males at 12–15 months of age (Diwan et al. 1997). Similarly, another laboratory reported liver tumors in 75% of naturally infected A/J males at 15–18 months of age (Fox et al. 1996a). Thus, the model seemed promising as an experimental system for study of bacterial carcinogenesis.

However, studies with experimental infection by injection of cultured bacteria have yielded lower liver tumor incidences. In an investigation involving ig treatment of mice with *H. hepaticus* at 10 weeks of age, and including both sexes and a selection of recombinant inbred strains, liver preneoplasia/neoplasia incidence at 14 months of age was, at most, 20% (Ihrig et al. 1999). Avenaud et al. (2003) exposed A/J mice at 3–4 weeks of age to a relatively low titer of *H. hepaticus* (10^7^) ig and examined small groups of mice at ages up to 17 months. Although all mice tested positive for the bacterium, hepatitis was relatively mild, and no liver tumors were observed. Possible insight into the reason for the low yield of liver tumors in these studies was offered by Rogers et al. (2004), who exposed A/J mice to *H. hepaticus* at various perinatal stages and at 12 weeks of age and evaluated the livers at time points up to 12 months of age. Although the limited numbers of mice in each group constrained statistical certainty, the results suggested that exposure before 12 weeks of age was required for development of hepatitis and liver dysplasia: none of the four male mice treated at 12 weeks had these lesions, whereas such changes occurred in 30–70% of those exposed by treatment of the dams at conception or on fetal day 10, or of offspring at 3 weeks of age.

We therefore tested whether progressive hepatitis and a high incidence of liver tumors with aging requires perinatal exposure, as would occur in naturally infected mouse colonies. Our finding that such is, in fact, the case may have bearing on the potential importance of human perinatal exposure to infectious agents on later health consequences. A preliminary report of this work has appeared in abstract form (Diwan et al. 2001).

## Materials and Methods

### H. hepaticus *culturing, treatment, and detection*

We used *H. hepaticus*, Frederick strain 1A (passage 7), which was isolated contemporaneously from the same colony of mice as those providing the tissues used for cloning and genetic analysis of the bacterium and deposition in the American Type Culture Collection (Fox et al. 1994, 1996b). We grew the bacteria under microaerophilic conditions at 37°C on plates of *Brucella* agar with horse blood and trimethoprim, vancomycin, and polymyxin B (TVP) (Remel Co., Lenexa, KS) for 3 days. This bacterial strain has subsequently been shown to be detected by *H. hepaticus*–specific polymerase chain reaction (PCR) primers and to be negative for all PCR-based tests for all other *Helicobacter* species. To check for motility and morphology, the organism was harvested, suspended in brain heart infusion broth with horse serum and yeast extract (Remel Co.), and visualized by phase-contrast microscopy.

For the *in vivo* studies, the organism was harvested and suspended in sterile phosphate-buffered saline (PBS), and the concentration was adjusted to a McFarland equivalence turbidity standard of 1.0 (3 × 10^8^ bacteria/mL). We tested viability of the suspended organisms by phase-contrast microscopy to determine motility and by subculturing onto *Brucella* blood agar plates with TVP as described above, both before and after animal inoculations.

All mice were obtained from the Animal Production Area at NCI Frederick. The National Cancer Institute (NCI)-Frederick is accredited by the Association for Assessment and Accreditation of Laboratory Animal Care International and follows the U.S. Public Health Service policy for the care and use of laboratory animals. We provided animal care in accordance with the procedures as outlined by the Institute of Laboratory Animal Resources (1996). Animals were treated humanely and with regard for alleviation of suffering. Animal treatments consisted of a single dose of 1.5 × 10^8^ bacteria in 0.5 mL PBS.

Treated mice were maintained in semi-rigid isolators (Charles River, Boston, MA), and controls were housed in rooms free of the bacteria. Mice were kept in polycarbonate cages with cellulose fiber chips as bedding. We assayed feces samples for the presence of *Helicobacter* spp. by passing the samples through a 0.45-μm filter and then streaking them onto *Brucella* agar with horse blood and TVP at 37°C under microaerophilic conditions. We compared appearance of growth of these cultures with that of starting cultures of *H. hepaticus*.

### Perinatal exposure by maternal injection with H. hepaticus

We used uninfected male and female A/JCr (A/J) mice for this study. We injected 4-week-old females ip or ig with 0.5 mL bacterial suspension. Feces samples from these mice were checked for presence of the bacteria after 3 weeks, and females positive for fecal bacteria were bred with uninfected males. We allowed some females to produce more than one litter. Male offspring were weaned and euthanized when moribund or at 18 or 24 months of age. Mothers were kept until euthanized because of morbidity. Female offspring were not retained because *H. hepaticus*–associated liver tumorigenesis has been observed mainly in male mice.

### Perinatal exposure by maternal and neonatal exposure to *H. hepaticus* through feces

Six cages containing 2-year-old A/J males with feces positive for *H. hepaticus* were available (one to three per cage). At the start, we removed the males from a cage that they had occupied for 4 days and transferred the soiled bedding to cages housing pregnant females (one or two such cages for each cage of males). Litters were born an average of 7 days after initial exposure to soiled bedding. This *H. hepaticus* exposure procedure was repeated daily, 5 days/week, until pups were weaned. We weaned 32 male offspring from nine litters and maintained them until moribund; remaining mice were euthanized at 24 months of age. We monitored feces from their cages periodically for the presence of *H. hepaticus*, collecting a total of eight samples over time from each cage.

Six of the male offspring from cages with positive tests for fecal *Helicobacter* were bred to uninfected females in order to test their capability for infection of subsequent generations. We obtained a total of 18 male F_2_ offspring.

### Experimental infection of weanling mice

Uninfected A/J males were treated at 3–4 weeks of age with *H. hepaticus* suspension ig or with PBS only. Mice exposed to the bacteria were maintained in isolators. At 2, 4, 12, 24, and 54 weeks after treatment, fecal samples were cultured to determine the presence of *H. hepaticus*, and mice from each group were euthanized as indicated in Table 1. Mice that died naturally or were moribund during the course of the study were included for analysis with the end point group closest in time. A small number of mice were allowed to live until natural morbidity; their average ages are given in Table 1. We collected livers from all animals up to 54 weeks and divided them into lobes; we either froze portions of each lobe in liquid nitrogen or fixed them in 10% neutral buffered formalin for histopathologic analysis. We analyzed two lobes each from randomly selected livers, representing each time point and group, to determine levels of 8-hydroxydeoxyguanosine (8-oxo-dG), a marker for oxidative DNA damage, in liver DNA as previously described (Sipowicz et al. 1997). Results for mice at 2, 4, and 12 weeks after exposure have been published previously (Sipowicz et al. 1997).

To test the effect of different genetic backgrounds, we performed a second experiment with male weanling mice of the BALB/cAnNCr (BALB/c), C57BL/6NCr (C57BL/6), and A/J strains. Small groups of mice were euthanized at 3, 6, and 12 months of age, and the remainder were maintained until morbidity or natural death. Fecal samples were monitored periodically for *Helicobacter*.

### Histopathology

We subjected all mice to complete necropsy and fixed tissues in 10% neutral formalin. Liver, stomach, and ceca, as well as any abnormal masses or lesions, were examined for each mouse. We evaluated severity of pathologic changes in livers semi-quantitatively on a scale of 1 to 4 (minimal, mild, moderate, or severe), and diagnosed typical *H. hepaticus*–associated hepatitis (chronic active hepatitis) on the basis of simultaneous presence of the following lesions: mild to severe multifocal necrosis, subacute inflammation, infiltration of peribiliary leukocytes, bile ductule hyperplasia, oval cell hyperplasia, hepatocytomegaly, and lymphocytic infiltration (Rice 1995; Ward et al. 1994a, 1994b). We also noted minimal foci of subacute inflammation and lymphocytic infiltrates, which are common liver lesions in normal mice of many strains, but they were not associated with *Helicobacter* infection and are thus not included here. Steiner’s silver stain was used for histologic confirmation of the presence of helical bacteria in liver sections.

### Statistical analysis

We analyzed data using GraphPad InStat, version 3.00 (GraphPad Software, San Diego, CA). Tests included a *t*-test for parametric data and Mann-Whitney or Kruskal-Wallis tests for nonparametric data as appropriate.

## Results

### Liver tumorigenesis in male A/J offspring after maternal exposure to *H. hepaticus*

Females treated with H. hepaticus suspensions ip and found to have positive fecal cultures were mated. Four females produced a total of 27 male offspring, which were housed in groups of two to four. All cages gave positive results for *H. hepaticus* in feces, and the number of mice per cage had no obvious relationship to outcomes (data not shown). At an average age of about 22 months, 11 (41%) of these male offspring had *Helicobacter* hepatitis. Of these, 9 (33%) had at least one liver tumor, 7 (26%) had multiple tumors, and 5 (18%) had hepatocellular carcinoma (Table 2). Hepatocholangiolar tumors, an adenoma and a carcinoma, occurred in 2 mice. We found tumors in offspring of each of the mothers. All of the hepatocellular tumors occurred in livers that also had *Helicobacter* hepatitis, which was moderate to severe in all cases except for one, which was categorized as mild. Three livers that did not have a tumor had minimal or mild chronic active hepatitis. The four mothers were euthanized when moribund at 49–87 weeks of age; of the four livers, three had chronic inflammation of moderate intensity, but no hepatitis characteristic of *Helicobacter* infection.

Female mice treated ig with the same *H. hepaticus* preparations as the ip-treated females and found to have positive fecal cultures were bred. Five mothers produced a total of 26 male offspring. Fecal cultures from both the mothers and the male offspring were positive for *Helicobacter*. Among these male offspring, 35% had *Helicobacter* hepatitis, an incidence similar to that in male offspring of the ip-treated females (Table 2). However, despite greater average age of these mice, the average severity of the hepatitis was less (close to significance). Only two mice (8%) had hepatocellular tumors, and no mouse had multiple liver tumors. These values are significantly different from those in the male offspring of ip-treated females. The five ig-treated mothers were euthanized at 67–105 weeks of age. We did not observe chronic inflammation of the liver (*p* = 0.048 compared with chronic inflammation in three of four ip-treated mothers).

To test the efficacy of bacterial transmission through feces, we exposed pregnant mice and their offspring up to the time of weaning to feces from *Helicobacter*-positive male mice. Eight litters yielded a total of 32 male offspring. Incidence of males with *Helicobacter* hepatitis (19%) was less than that in offspring of mothers exposed ip (41%), but this difference was only of borderline significance (*p* = 0.086), and average severity grade was similar (Table 2). We observed only one liver tumor, a hepatocellular carcinoma in a male of advanced age without hepatitis. Feces from four of the nine cages housing these mice were positive for *H. hepaticus* at approximately monthly samplings between 1 month and 1 year of age.

We further tested fecal transmission by mating *Helicobacter*-positive male offspring from the above study with uninfected females. We obtained a total of 20 male off-spring from nine litters. Although these F_2_ mice were terminated at earlier ages than mice in the other studies, the incidence and average grade of *Helicobacter* hepatitis were very similar to those for the F_1_ males exposed directly to contaminated feces during gestation and infancy (Table 2). We found no liver tumors in the F2 males.

We noted typhlitis and hyperplasia of gut-associated lymphoid tissue (GALT) in 4–19% of the male offspring in the various perinatal exposure groups, with no significant differences among them.

### Lack of tumorigenic effectiveness of ig treatment of A/J weanling mice with H. hepaticus

We tested *H. hepaticus* of the same strain used for the perinatal studies above in weanling A/J mice. Chronic active hepatitis associated with experimental ig *H. hepaticus* infection was first apparent after 12 weeks after exposure and occurred with low frequency at later time points, but never exceeding 20% (Table 1). We noted mild-to-moderate histopathologic changes (scores of 2–3) in 6 of 15 (40%) of the livers of experimentally infected mice at 12 weeks after exposure but none in the controls (*p* = 0.0068). Subacute inflammation was the most common hepatic lesion (5 of 15) in these mice. The incidence of this change decreased at 24 weeks (1 of 10) and 36 weeks (1 of 15) after exposure. We observed mild-to-moderate peribiliary leukocyte infiltration in a small percentage of the exposed mice at ≥ 12 weeks after exposure, but in none of the controls through 54 weeks (4 of 45 vs. 0 of 47, *p* = 0.053). Similarly, mild-to-moderate necrosis occurred only in the exposed mice (9 of 81 vs. 0 of 82, *p* = 0.0015).

Thus, we observed apparent hepatic effects of the *H. hepaticus* infection in some of the mice, but these were not striking, and typical *Helicobacter*-associated hepatitis was rare. Correspondingly, only two mice, one exposed and one control, presented with liver tumors at advanced ages. The control tumor, at 751 days of age, was a hepatocellular adenoma. An exposed mouse at 790 days of age presented four hepatocellular adenomas, one hepatocholangiolar adenoma, and a hemangiosarcoma within the liver; however, hepatitis typical of *H. hepaticus* was not present.

We measured levels of 8-oxo-dG indicative of oxidative DNA damage in two to five randomly selected mice for each treatment category and time point up to 1 year; in most cases, samples from the median and left liver lobes were analyzed for each mouse. As shown in Table 1, a significant increase in hepatic 8-oxo-dG had already occurred within 2 weeks of infection. We obtained very similar results at 4 weeks. We noted a further increase at 12 weeks after exposure, where the elevated level of 8-oxo-dG was highly significant compared with controls at this age and compared with exposed mice at 4 weeks. Thereafter, the level of 8-oxo-dG in the livers of the infected mice did not change significantly, whereas that in the controls increased steadily, as is expected with aging. The difference relative to the controls was still significant at 24 weeks after exposure, but not at 36 or 54 weeks. 8-oxo-dG levels were not clearly correlated with histopathologic findings: At 24 weeks postexposure, the average number of 8-oxo-dG residues in hepatic DNA from the mouse with clear *Helicobacter* hepatitis was 2.27/10^5^ dG residues, compared with 2.28/10^5^ dG in one without liver lesions. At 54 weeks after exposure, the liver with hepatitis had 1.67 8-oxo-dG residues per 10^5^ dG, whereas one with minimal hepatic change had 1.74 residues/10^5^ dG. However, because of the focal nature of the hepatitis, lack of correspondence with 8-oxo-dG levels cannot be concluded with certainty.

Fecal samples taken from cages of experimentally infected mice at 2–24 weeks after exposure were positive for *H. hepaticus*. No samples were taken at 36 weeks. For the three cages of mice at 54 weeks after exposure, two cages were positive and one was negative. No bacterial growth occurred for the control fecal samples at any time point.

With regard to the gastrointestinal tract, we found hyperplasia of the glandular stomach in 17 of 81 (21%) exposed mice and in 11 of 82 (13%) controls, not a significant difference (*p* = 0.22). Histopathologic examination of ceca revealed hyperplasia of GALT in 7–40% of mice, with no significant differences in incidence between exposed and control mice. Proliferative typhlitis occurred predominately in the exposed mice (Table 1); incidence was highest at 12–54 weeks after exposure and was significantly greater than controls at 12, 24, and 36 weeks, with an apparent decrease thereafter. The incidence in all exposed mice at 12–36 weeks (21 of 40) was significantly greater than the incidence at > 54 weeks (0 of 6; *p* = 0.025).

### Confirmation of lack of tumorigenic effectiveness of experimental infection with *H. hepaticus* in A/J, BALB/c, and C57BL/6 as weanlings

To confirm the results noted above for A/J mice and to test whether other mouse strains might be more susceptible, we repeated the experiment and included BALB/c and C57BL/6 mice (Table 3). Small subsets of mice were euthanized at interim time points and all mice remaining at 1 year of age were maintained until natural morbidity. Fecal samples throughout the course of the study confirmed the presence of helical bacteria shed from all of the treated mice and from none of the controls. We observed few age-dependent differences. A summary of lesions for all mice in the study is presented in Table 3.

We diagnosed chronic active hepatitis rarely in two exposed BALB/c mice and one exposed C57BL/6 mouse (Table 3). Liver tumors occurred in low incidence in old mice and were not significantly different in treated versus control groups. Among the experimentally infected mice presenting *H. hepaticus*–associated hepatitis, we found one hepatocellular carcinoma in a BALB/c mouse at 778 days of age, and three adenomas in a C57BL/6 mouse at 720 days.

Several histopathologic changes were significantly associated with treatment in at least one of the mouse strains. We found hyperplasia of the nonglandular stomach in 6 of 30 (20%) infected A/J mice, compared with 0 of 29 controls (*p* = 0.024). Five of the cases were in old mice, and four were moderate or severe. In the other strains, gastric hyperplasia was uncommon and not associated with treatment. Table 3 shows other significant alterations. In A/J mice, proliferative typhlitis was more common in ceca of infected versus control mice at all end points, and these differences were significant for all mice combined (Table 3). However, the incidence of proliferative typhlitis was reduced to 7% in infected A/J mice at 2 years of age. Significant differences in these parameters were also observed for BALB/c mice; in this strain, frequency of hyperplasia of GALT was also higher in the mice infected with *H. hepaticus*: 15 of 23 (65%) versus 9 of 33 (27%) (*p* = 0.0066).

Fecal samples confirmed that all three strains of experimentally infected mice, but no controls, were shedding *H. hepaticus* at all time points.

## Discussion

The results of this study confirm the findings of other investigations (Avenaud et al. 2003; Ihrig et al. 1999); we found that ig injection of weanling or young adult A/J mice with *H. hepaticus* reliably leads to early hepatic changes—in 40% of the mice in our study at 12 weeks after exposure—as well as marked increases in oxidative DNA damage, but liver tumors are rarely an outcome. With increasing time after exposure, fecal samples became less uniformly positive, a lower proportion of livers exhibited *Helicobacter*-related damage, and the elevated oxidative DNA damage relative to controls disappeared. We discovered few hepatitis-related tumors or preneoplastic foci, none in A/J mice. These results are in contrast to those reported for naturally infected mice; for example, Sipowicz et al. (1997) measured a steady increase in 8-oxo-dG at 10, 12, and 18 months of age. Such mice had high incidence (92%) of liver tumors at 18 months of age (Ward et al. 1994b). Together, the results are consistent with host immune response successfully suppressing *H. hepaticus* in mice infected after weaning.

In the A/J and BALB/c mouse strains, infection with *H. hepaticus* resulted in several hepatic and cecal changes, including proliferative typhlitis, as observed in other studies. We observed no effects in C57BL/6 mice, confirming the resistance of this strain to hepatic effects of the bacteria (Ihrig et al. 1999; Ward et al. 1994a, 1994b), despite the greater concentration of bacteria in the ceca of C57BL/6 than in A/J mice (Whary et al. 2001).

In contrast, perinatal infection of A/J mice by ip injection of their mothers with *H. hepaticus* before breeding led to a high incidence of progressive hepatitis and significant numbers of multiple liver tumors, including hepatocellular carcinomas, in their male offspring. This confirms the previous finding of Rogers et al. (2004). The present study partially reproduces the high incidence of liver tumors in naturally infected A/J males and provides an experimental paradigm for generating *H. hepaticus*–associated liver tumors. We observed lesser effects in male offspring of mothers treated with *H. hepaticus* ig before conception. Intragastric treatment might be expected to be less effective for establishing a systemic infection with *H. hepaticus* than ip treatment. We confirmed this by the presence of chronic inflammation in the livers of ip-treated mothers but not ig-treated mothers. In a study with *H. pylori* and a novel *Helicobacter* species, McCathey et al. (1999) found that ip-administered bacteria, but not ig administration at the same titer, resulted in hepatitis and elicited a much greater immune response. Thus, the likelihood of transmission of progressive disease to offspring may be a function of degree of infection of the mother, and the very high incidences of hepatitis and tumors in male offspring of naturally infected mothers, seen in our original population of infected mice, may reflect the level of bacterial burden accumulated over generations. However, the possibility of attenuation of bacterial virulence with serial passaging cannot be ruled out at present.

The bacteria might pass from mother to offspring transplacentally, through milk, or through feces. Li et al. (1998) were able to culture *H. hepaticus* from about 20% of fetal viscera in severe combined immunodeficiency (SCID) mice, but not from any of 14 A/J fetuses. This indicates that transplacental transmission is at least possible. However, Singletary et al. (2003) eliminated mother-to-pup transmission of *H. hepaticus* for C57BL/6 females experimentally infected ig with this bacterium by foster nursing the newborns within 24 hr after birth. Thus, under these conditions, at least, transmission was not transplacental. In the present study, establishment of the infection by exposure to feces from infected males, although resulting in hepatitis in some mice, did not lead to tumors, but maternal feces might be more effective. Passage of bacteria in the milk is theoretically possible.

The mechanism of hepatic tumorigenesis after perinatal exposure may well involve immune tolerance. In genetically engineered mice heterozygous for the *Apc* gene (*Apc*^Min/+^) and deficient in lymphocytes because of lack of *Rag2*, *H. hepaticus* infection increased both colon and mammary tumor incidence (Erdman et al. 2003; Rao et al. 2006a, 2006b). This tumorigenic effect was suppressed by CD4^+^CD45RB^lo^CD25^+^ regulatory T cells, especially if these were obtained from *H. hepaticus*–infected mice. Such regulatory T cells have a role in establishing tolerance to self-antigens, which evolves during the neonatal period (e.g., Arnold et al. 2005). One hypothesis is that *H. hepaticus* or a bacterial product present during the neonatal period becomes identified as “self” and so escapes effective immune surveillance. An interesting feature in the perinatal models we used in our experiments was that hepatitis occurred to similar degrees with all four modes of infection, but hepatic tumor outcomes were quite different. Thus, tumorigenesis involves something more than hepatitis. *H. hepaticus* expresses a cytolethal distending toxin (CDT) (Chien et al. 2000), which is essential for colonization of the intestines of normal mice (Ge et al. 2005), as well as for severe typhlocolitis in immunodeficient mice (Young et al. 2004). CDT suppresses host immune response to the bacteria (Pratt et al. 2006); both T cells and antigen-presenting cells may be affected. Of particular interest in the present context, when Ge et al. (2007) exposed A/J mice to a high titer of bacteria, both wild-type and CDT mutant *H. hepaticus* resulted in comparable degrees of hepatitis at 4 and 10 months of age, but only mice infected with the wild-type bacteria showed hepatic preneoplastic foci. A number of proinflammatory mediators, including tumor necrosis factor −α, interleukin-6, and transforming growth factor −α, were reduced in the absence of CDT.

These results confirm that hepatitis and tumorigenesis are separable, and link CDT specifically to the tumor development process through its effects on immune system balance. It may be that immune response to CDT specifically is suppressed during the acquisition of immune tolerance in the neonatal period, resulting in higher levels of CDT production, a qualitatively and/or quantitatively enhanced proinflammatory, protumorigenic environment, and ultimately liver tumor development. Chronic inflammatory events have long been associated with the promotion phase of liver tumorigenesis in rodents. Promotion of liver tumors by *H. hepaticus* infection has been demonstrated (Diwan et al. 1997).

Furthermore, the CDT also causes DNA damage by its DNase activity (Avenaud et al. 2004) and thus could contribute to tumor initiation. It is possible that this property of CDT explains the special sensitivity of perinates, because liver tumors are initiated by genotoxicants with higher efficiency in neonatal than in adult rodents (reviewed by Anderson et al. 2000). In this scenario, chronic hepatitis from *H. hepaticus* might lead to liver tumors effectively only when these have been initiated perinatally. These hypotheses may be readily tested with the model that we have established. In view of the enhanced virulence of infectious agents encountered in early life in humans, with regard to hepatic cancer caused by hepatitis viruses (Stuver 1998), and gastric cancers related to *H. pylori* (Blaser et al. 1995) and possibly to Epstein-Barr virus (Abdirad et al. 2007), pursuit of these mechanisms is important.

## Figures and Tables

**Table 1:** Hepatic and cecal lesions and hepatic oxidative DNA damage in A/J mice exposed ig as weanlings to *H. hepaticus* and in controls

					Oxidative DNA damage^a^		
Weeks after exposure	Group	No.	Tumors [*n* (%)]	Chronic active hepatitis [*n* (%)]	No.^b^	8-Oxo-dG/10^5^ dG ± SE	Cecal proliferative typhlitis [No. (%), severity score^c^]
2	Exp	15	0	0	3 (6)	1.3 ± 0.1*	1 (7), 1.0
	Con	15	0	0	3 (4)	0.8 ± 0.1	0
4	Exp	15	0	0	3 (6)	1.3 ± 0.1**	2 (13), 1.0 ± 0
	Con	16	0	0	2 (4)	0.8 ± 0.03	1 (7), 1.0
12	Exp	15	0	2 (13)	3 (6)	2.1 ± 0.2**	6 (40)*, 1.7 ± 0.2
	Con	16	0	0	3 (5)	1.2 ± 0.1	1 (6), 1.0
24	Exp	10	0	1 (10)	5 (9)	2.3 ± 0.1**	7 (70)**, 1.6 ± 0.3
	Con	10	0	0	5 (10)	1.5 ± 0.1	0
36	Exp	15	0	0	4 (7)	2.1 ± 0.3	8 (53)**, 1.6 ± 0.2
	Con	16	0	0	4 (8)	2.0 ± 0.2	1 (6), 1.0
54	Exp	5	0	1 (20)	5 (10)	2.3 ± 0.3	2 (40), 2.0 ± 0
	Con	5	0	0	5 (10)	2.0 ± 0.1	0
> 54^d^	Exp	6	1 (17)	0	ND		0
	Con	4	1 (25)	0	ND		0

Abbreviations: Con, untreated controls; Exp, experimentally infected with *H. hepaticus*; ND, not done

a Results for weeks 2–12 were reported previously by Sipowicz et al. (1997).

b Number of livers analyzed (total number of samples).

c Mean ± SE.

d Mean (± SE) ages of experimental and control mice were 743 ± 48 and 623 ± 68 days, respectively.

* *p* < 0.05, and

** *p* < 0.01, compared with control mice at the same time point.

**Table 2:** Histopathologic findings in A/J mice exposed to *H. hepaticus* perinatally and in controls [*n* (%)].

Characteristic	Mothers exposed ip	Mothers exposed ig	Mothers and newborns exposed through feces	F_2_ offspring of feces-exposed males
No.	27	26	32	20
Average age (days)	674 ± 18	674 ± 28	647 ± 22	458 ± 16
*Helicobacter* hepatitis positive	11 (41)	9 (35)	6 (19)	5 (25)
Average grade	3.0 ± 0.4*	2.0 ± 0.3*	2.5 ± 0.2	2.6 ± 0.6
Average age (days)	669 ± 20	713 ± 18	675 ± 62	441 ± 40
Hepatocellular tumors present	9^a^ (33)**, #	2 (8)**	1 (3)#	0
Multiple hepatocellular tumors^b^	7 (26)##,†	0##	0†	0
Hepatocellular carcinoma	5 (18)	1 (4)	1 (3)	0
Typhlitis	1 (4)	3 (12)	3 (9)	1 (5)
Hyperplasia of GALT	1 (4)	3 (12)	6 (19)	3 (15)

Values shown are mean ± SE or number (%) except where indicated.

a Eight had hepatocellular tumors (three with adenomas only, five with adenoma and carcinoma, one each with hepatocellular adenoma and carcinoma and either cholangioma or cholangiocarcinoma), and one had cholangioma only. The liver with hepatocholangiolar adenoma was the only liver with tumor that did not have *Helicobacter* hepatitis.

b Multiplicity could not be quantified because of extensive tumor involvement throughout many of the livers. Significance of differences between values with matched superscripts:

* *p* = 0.07,

** *p* = 0.039,

# *p* = 0.0035,

## *p* = 0.01,

† *p* = 0.0026

**Table 3:** Summary of lesions in livers and ceca of mice of different strains experimentally infected as weanlings with *H. hepaticus* and in controls.

Strain	Group	No.	Hepatitis	Liver tumor	Proliferative typhlitis
A/J	Infected	30	0	0	11 (37)*
	Control	29	0	1^a^	3 (10)
BALB/c	Infected	23	2 (9)	1 (4)^b^	7 (30)*
	Control	33	0	3 (9)^c^	1 (3)
C57BL/6	Infected	33	1 (3)	6 (18)^d^	1 (3)
	Control	28	0	8 (29)^e^	3 (11)

Values shown are number (%).

a Hepatocellular adenoma, mouse 462 days of age.

b Hepatocellular adenoma, mouse 778 days of age, grade 2 hepatitis; also eosinophilic foci.

c Two hepatocellular adenomas in mice 637 and 683 days of age, and one carcinoma in mouse 610 days of age; all without hepatitis.

d Three mice with single adenomas, one mouse with three adenomas and hepatitis (negative for presence of *Helicobacter* with Steiner’s stain), and one mouse each with hepatoblastoma or with hepatocholangiocarcinoma.

e Four mice with single hepatic adenoma, two mice each with two adenomas, and two mice each with an adenoma and a carcinoma.

* *p* < 0.05 compared with control mice of that strain.
